# The Viremic Phase and Humoral Immune Response Against African Horse Sickness Virus That Emerged in Thailand in 2020

**DOI:** 10.3390/vetsci12090878

**Published:** 2025-09-11

**Authors:** Paphavee Pipitpornsirikul, Nattarat Thangthamniyom, Aree Laikul, Tapanut Songkasupa, Watcharapol Pathomsakulwong, Tawanhathai Apichaimongkonkun, Suwicha Kasemsuwan, Teerasak E-kobon, Porntippa Lekcharoensuk

**Affiliations:** 1 Center for Agricultural Biotechnology, Kasetsart University, Kamphaeng Saen Campus, Nakhon Pathom 73140, Thailand; paphavee.pi@ku.th; 2 Center of Excellence on Agricultural Biotechnology: (AG-BIO/MHESI), Bangkok 10900, Thailand; 3 Department of Microbiology and Immunology, Faculty of Veterinary Medicine, Kasetsart University, Bangkhen Campus, Bangkok 10900, Thailand; nattarat.tha@cpf.co.th; 4 Department of Large Animal and Wildlife Clinical Science, Faculty of Veterinary Medicine, Kasetsart University, Kamphaeng Saen Campus, Nakhon Pathom 73140, Thailand; fvetarl@ku.ac.th; 5 Virology Section, National Institute of Animal Health, Department of Livestock Development, Bangkok 10900, Thailand; tapanut.s@dld.go.th; 6 Equine Clinic, Kasetsart University Veterinary Teaching Hospital, Kasetsart University, Kamphaeng Saen Campus, Nakhon Pathom 73140, Thailand; watgolf2000@hotmail.com (W.P.); Tawanhathai.ap@ku.th (T.A.); 7 Department of Veterinary Public Health, Faculty of Veterinary Medicine, Kasetsart University, Kamphaeng Saen Campus, Nakhon Pathom 73140, Thailand; fvetswk@ku.ac.th; 8 Department of Genetics, Faculty of Science, Kasetsart University, Bangkhen Campus, Bangkok 10900, Thailand; teerasak.e@ku.th

**Keywords:** African horse sickness virus, immunity, vaccine, viremia, disease control

## Abstract

African horse sickness (AHS), a life-threatening disease caused by African horse sickness virus serotype 1 (AHSV-1), emerged in Thailand in February 2020 with 607 cases and a 93% fatality rate. The outbreak was mitigated by vector control and a live attenuated virus (LAV) vaccine. Information regarding viremia and immunity after infection and vaccination during outbreaks are essential for controlling disease transmission. This study evaluated these parameters in 15 infected naïve horses and 11 vaccinated horses during the 2020 outbreak. Whole blood was collected and subjected to RT real-time PCR to detect viremia up to 52 weeks post-infection. ASHV-specific antibodies were accessed by blocking ELISA until 37 weeks post-vaccination. In both groups, viremia appeared in the first week and lasted until week 5 in 25% of the horses. Moreover, seroconversion occurred at week 5 in 73.3% of infected horses and at week 3 in 90% of vaccinated horses. Antibodies persisted in both groups until the end of study, with more uniform levels in vaccinated animals. Chronological relations among clinical signs, viremia, and immunity were analyzed. These findings support the use of insect-proof housing for horses for at least 5 weeks after exposure or vaccination, and provide essential insights into AHSV epidemiology and control.

## Simple Summary

In 2020, African horse sickness (AHS), a deadly horse disease caused by African horse sickness virus (AHSV), appeared in Thailand for the first time, infecting 607 horses, 93% of whom died. The outbreak was controlled by vaccinating healthy horses with a live attenuated virus vaccine and confining infected and vaccinated horses separately in insect-proof housing. However, little was known about how long the virus stayed in the blood and how horses’ immune systems responded after infection or vaccination during the outbreak. Here, we examined 15 naturally infected and 11 vaccinated horses in Thailand by collecting their blood and serum samples after infection and vaccination during the outbreak. The virus was detected in about 25% of horses in both groups up to week 5. Most infected and vaccinated horses developed antibodies by weeks 5–6 and week 3, respectively. Antibodies remained for at least 12 months in infected horses and 8 months in vaccinated horses. These findings provide important information for AHS prevention and control and support the protection of horses using insect-proof housing for at least 5 weeks after AHSV exposure or vaccination.

## 1. Introduction

African horse sickness (AHS) is an equine infectious and arthropod-borne disease caused by African horse sickness virus (AHSV). Among equids, horses are the most susceptible animals to the disease; they develop severe clinical signs after infection and exhibit a mortality rate of 70–95%. Mules and donkeys are less susceptible, while infected zebras and African donkeys show only subclinical signs [1]. Currently, no specific treatment is available for AHS; therefore, supportive care is the only therapeutic option. In recovered animals, clinical signs usually subside within 7 days [2]. AHS is transmitted by infected midge bites; *Culicoides* spp., mainly *C. imicola* and *C. bolitinos*, are the primary vectors [3]. AHS causes high mortality in horses and is rapidly transmitted throughout the world, resulting in severe losses to the equine industry and economy and significant impacts on human mental health. Therefore, control and prevention of the disease are critical. In addition, this disease is included on the World Organisation for Animal Health (WOAH) list of terrestrial animal diseases [4].

African horse sickness virus (AHSV) is a non-enveloped, double-stranded RNA virus classified in the genus *Orbivirus*, of the family *Reoviridae*. Its genome comprises 10 linear segments encoding seven structural proteins (VP1–7) and five non-structural proteins (NS1, NS2, NS3/3a, and NS4). The outer capsid consists of VP2 (Segment 2) and VP5 (Segment 6). As the most variable protein, VP2 determines the virus serotype, allowing classification into nine serotypes (AHSV-1 to -9). Therefore, it is used for serotype-specific differentiation in diagnostic laboratories. In contrast, VP7 (Segment 7), a highly conserved inner capsid protein, is commonly used for group-specific detection [5].

Although endemics of AHS had occurred in African countries, WOAH reported a new AHS outbreak area outside Africa, caused by AHSV serotype 1 (AHSV-1), as the first incident of an AHS outbreak in Southeast Asia [6]. AHS was first recognized in Thailand in February 2020 and officially announced by WOAH on 27 March 2020. The disease rapidly spread to 17 provinces of Thailand and affected more than 2000 horses, with 607 sick animals and 500 deaths, leading to a case fatality rate of 93% [7]. During the outbreak, the Department of Livestock Development (DLD) of Thailand implemented various control measures to cease the spread. These included confining healthy and AHSV-positive horses separately in different insect-proof net stables and recommending insect repellent for outdoor horses and horse nets. Moreover, all susceptible animals in the outbreak areas were tested to ensure that they were ASHV-negative before being immunized with a single dose of the live attenuated AHSV vaccine (AHSV-LAV) combination 1 containing AHSV serotypes 1, 3, and 4. After immunization, horses were housed in a stable with an insect-proof net for 30 days to prevent vector exposure and allow the development of immunity. The recovered and non-infected horses could be regrouped once they were confirmed to be AHSV-negative. Consequently, the disease was eradicated after the last recorded case in September 2020 [6]. Thailand finally regained its status as an AHS-free country from WOAH on 10 March 2023 [8].

The duration of viremia and the immune response following AHSV infection or vaccination are key factors not only for understanding disease progression and assessing vaccine efficacy, but also for determining appropriate measures for disease control and prevention. In AHSV-infected horses, viremia typically lasts for 4–8 days, but can extend up to 21 days [9]. Additionally, a previous study involving experimental AHSV infection demonstrated that antibody responses were detectable from days 14 to 56, which marked the end of the study period [10]. In contrast, another study reported that viremia persisted until week 12 post-vaccination in horses immunized with AHSV-LAV combination 1, prior to receiving combination 2 [11]. Moreover, vaccination with AHSV-LAV combination 1 alone in horses could induce long-duration immunity from 1 month to at least 12 months after vaccination [12]. However, the kinetics of viremia and the immune response after the first AHSV infection during outbreaks has never been documented. In addition, information regarding viremia after a single dose of the AHSV-LAV combination 1 vaccination in horses raised in Southeast Asia is limited. Herein, we determine the kinetics of viral infection and the immune response in naturally infected naïve horses and single LAV-vaccinated horses in Thailand. This information is a key component for improving AHS prevention and control measures in the future.

## 2. Materials and Methods

### 2.1. Study Design and Sample Collection

This study was conducted during the AHSV outbreak that lasted from 25 March 2020 until December 2022. Viral detection was performed in a biosafety level 2 enhanced laboratory, and the animal experimental protocol was approved by the Institutional Animal Care and Use Committee of the Faculty of Veterinary Medicine, Kasetsart University, Bangkok, Thailand, under approval number ACKU67-VET-013. The owners of the studied horses were informed regarding the study aims, as well as animal handling and sample collection during the outbreak, in accordance with this protocol. The study’s focus was divided into two specific aims: (1) genetic characterization to identify the most divergent gene between the AHSV-1 wild type and vaccine strains that could distinguish naturally infected from vaccinated horses; (2) investigation of viral and antibody kinetics in naturally AHSV-infected naïve horses and AHSV-vaccinated horses.

**Figure 1:**
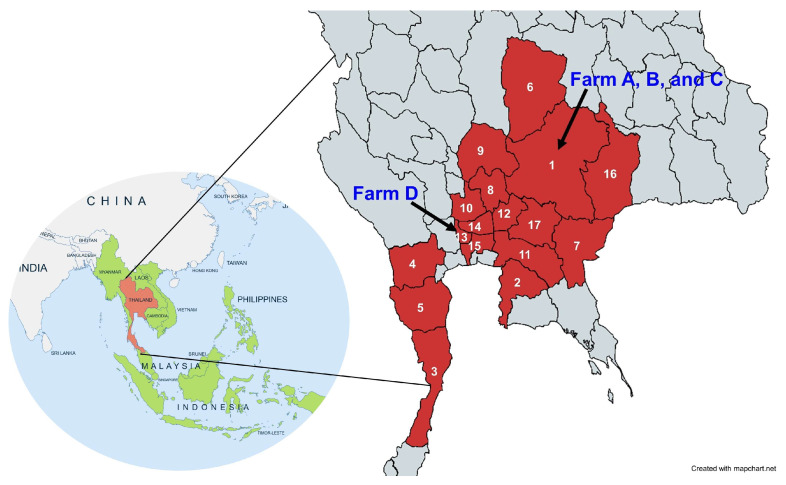
AHS outbreak areas in Thailand (red) and the locations of the studied farms (arrows). As of October 2020, the disease spread to 17 provinces, including Nakhon Ratchasima (1), Chonburi (2), Prachuap Khiri Khan (3), Ratchaburi (4), Phetchaburi (5), Chaiyaphum (6), Sa Kaeo (7), Saraburi (8), Lop Buri (9), Phra Nakhon Si Ayutthaya (10), Chachoengsao (11), Nakhon Nayok (12), Nonthaburi (13), Pathum Thani (14), Bangkok (15), Buriram (16), and Prachin Buri (17) [6]. The naturally AHSV-infected farms (A–C) were in Nakhon Ratchasima, and the vaccinated-horse farm was in Nonthaburi (D).

To obtain an AHSV-positive sample for the subsequent genetic characterization and assay development, a whole blood sample was collected from an AHSV-infected horse (AHSV2020/043) in Chachoengsao province, one of the outbreak areas in Thailand. This horse showed clinical signs of depression, supraorbital fossa edema, and conjunctival congestion, and it died shortly. For viral and antibody kinetics studies in naturally AHSV-infected horses, 15 horses from three infected farms located in Nakhon Ratchasima (Farms A–C), a province in Northeastern Thailand (Figure 1 and Figure S1), were included in the naturally AHSV-infected naïve horse group. These horses were confirmed to be infected with AHSV by RT-PCR conducted at the National Institute of Animal Health (NIAH), Thailand, and the Kamphaeng Saen Veterinary Diagnostic Center (KVDC), Faculty of Veterinary Medicine, Kasetsart University. All the horses had clinically recovered, had not been vaccinated against AHSV, and were housed in insect-proof stables to prevent virus transmission and re-infection throughout the outbreak. It is important to note that none of the infected horses were vaccinated, in accordance with the DLD’s policy. EDTA blood and serum samples were collected from each horse by jugular venipuncture. For viral detection, EDTA blood samples were collected for viral detection from the 15 AHSV-infected horses on days 2–3; subsequently at weeks 1, 3, and 5; and then during weeks 8–9, 12–14, 16–18, 20–22, 24–26, 30–31, 35–37, 39–41, 43–45, 47–49, and 51–53 after the index case was identified at each farm (Figure S2). Serum samples were obtained from each AHSV-infected horse on the same day as collecting the EDTA blood. Day 0 of antibody response was the day that the viral infection was confirmed by VP7-specific RT-PCR (Figure S2). Unfortunately, the EDTA blood samples of 10 horses on Farm C, which were intended for use in viral detection, were unsuitable for testing due to freezer failure. As a result, only EDTA samples from five horses on two infected farms were available for the viremia analysis. However, sera from all 15 horses were available and were used for the antibody response study as planned.

To study viremia and humoral immune response in post-vaccination horses, blood samples were collected from 11 horses on an AHSV-free farm in Nonthaburi (Farm D), a province in the Central Thailand (Figure 1). All horses were vaccinated once by subcutaneous injection with AHSV-LAV combination 1, which contains AHSV serotypes 1, 3, and 4 (Onderstepoort Biological Products; OBP, Pretoria, South Africa). The horses stayed in their stable with insect-proof nets for 30 days after vaccination. EDTA and whole blood samples were obtained from the 11 vaccinated horses before vaccination (D0), weekly from week 1 to 7, and then every other week at weeks 9, 11, 13, and 15 after vaccination. Sera were also obtained at weeks 19, 25, 31, and 37, the last week before the booster program.

These time points for sample collection were selected to monitor the kinetics of both viremia and humoral immune response, covering the acute phase and long-term monitoring after natural infection or vaccination, while minimizing the frequency of handling and blood collection, in accordance with animal welfare considerations. The horses’ clinical signs after infection and vaccination were also recorded throughout the study.

The collected EDTA blood samples were centrifuged at 1000× *g* at 4 °C for 10 min. The whole blood (0.5 mL) was transferred to a clean microfuge tube, mixed with 1.5 mL TRizol reagent (Invitrogen, Carlsbad, CA, USA), and stored at −80 °C until used for RNA extraction. The clotted blood samples were centrifuged at 2000 rpm at 4 °C for 15 min. Then, 1 mL of serum was transferred to a clean microfuge tube and stored at −20 °C until use for antibody testing.

### 2.2. RNA Isolation and cDNA Synthesis

The whole blood samples lysed in TRizol reagent were subjected to RNA extraction and cDNA synthesis. We extracted RNA from 800 µL of each sample using the Direct-zol™ RNA MiniPrep kit (ZYMO RESEARCH, Irvine, CA, USA), following the manufacturer’s instructions. This RNA was then used as the template for cDNA synthesis using the RevertAid RT Reverse Transcription kit (Thermo Fisher Scientific, Waltham, MA, USA). First, a 12.5 µL reaction volume of the RNA template and a random hexamer primer was incubated at 65 °C for 5 min. The reaction was then chilled on ice before adding 7.5 µL of a mixture of dNTPs, RNase inhibitors, and an enzyme. The total reaction volume of 20 µL of contained 4 µL of 50 ng/µL random hexamers (Invitrogen), 4 µL of 5× reaction buffer, 2 µL of 10 mM dNTP mix (Biotechrabbit™, Berlin, Germany), 0.5 µL of 40 U/µL RiboLock RNase inhibitors (Thermo Fisher Scientific, Waltham, MA, USA), and 1 µL of 200 U/µL reverse transcriptase (Thermo Fisher Scientific) combined with 8.5 µL of the extracted RNA template. The cDNA synthesis was performed in a thermal cycler (Mastercycler^®^ nexus GSX1, Eppendorf, Hamburg, Germany) by setting the temperature at 25 °C for 5 min followed by 42 °C for 60 min. The reaction was terminated by incubation at 70 °C for 5 min. The synthesized cDNA was chilled on ice for the next step or stored at −20 °C until used.

### 2.3. AHSV Detection Using Group-Specific (VP7) RT-PCR and RT Real-Time PCR

Initially, conventional gel-based RT-PCR was used to identify AHSV VP7 in the whole blood sample from the AHSV2020/043 isolate using a set of primers specific to the VP7 gene (Table 1). RT-PCR was performed as described elsewhere [13]. Briefly, the PCR reaction was composed of 2 µL of cDNA template, 2 mM dNTP mix (Biotechrabbit™), 2 µM each of forward and reverse primers, 1× (NH_4_)_2_SO_4_ buffer, 20 mM MgCl_2_, 0.25 U/µL Taq DNA polymerase (Thermo Fisher Scientific, Waltham, MA, USA), and RNase/DNase-free water to make the volume up to 10 µL. The PCR was performed in a thermal cycler (Mastercycler^®^ nexus GSX1, Eppendorf, Hamburg, Germany) with the following thermal profile: 94 °C for 3 min for initial denaturation, then 35 cycles of 94 °C for 30 s, 56 °C for 30 s, and 72 °C for 40 s, followed by a final extension step at 72 °C for 7 min. The amplified PCR products were confirmed in 1.2% agarose gel by gel electrophoresis. A sample showing a 265 bp DNA band was considered AHSV-positive.

RT real-time PCR was later utilized for AHSV identification and quantification from naturally infected naïve horses and AHSV-LAV combination-1-vaccinated horses using the same primers. A ten-fold serial dilution of a plasmid containing the VP7 gene of the AHSV vaccine strain (in-house preparation) was used to plot a standard curve that could be used for plasmid copy number determination to quantify AHSV in the sample. RT real-time PCR was conducted in accordance with the manufacturer’s instructions. A 10 µL PCR reaction, comprising 1× SSoFast EvaGreen supermix (Bio-Rad, Hercules, CA, USA), 2 μM forward and reverse primers (similar to those used for the above VP7 specific conventional RT-PCR), 2 μL of RNase/DNase-free water, and 2 μL of cDNA template, was performed in a real-time thermal cycler (Bio-Rad’s CFX96, Hercules, CA, USA). The thermal profile was 95 °C for 30 s, then 40 cycles of 95 °C for 5 s, followed by 65 °C for 5 s. For the melting curve analysis, the temperature was slowly increased from 65 to 95 °C (in 0.5 °C inc.) for 10 s/step. A sample was classified at 1000 RFU as AHSV-positive when its Ct value was less than 35 PCR cycles, and as AHSV-negative when its Ct value was over 35 PCR cycles [14]. The Tm value from a single melt peak associated with the amplification product is 82.50 °C (±0.5). The amplicon of 265 bp was confirmed by gel electrophoresis.

### 2.4. VP5 Gene Sequencing and Phylogenetic Analysis

The nucleotide sequences of all 10 gene segments of the AHSV-1 Thai strain, retrieved from the GenBank database, were compared with those of all three serotypes of AHSV-LAV combination 1 using MegAlign software (Lasergene, DNASTAR Version 11.1.0), in order to identify the most divergent gene. Among all the segments analyzed, VP5 exhibited the highest degree of nucleotide divergence. Therefore, it was selected as the target gene for assay development to differentiate between the field and vaccine strains [13]. In addition, the VP5 gene of Thai AHSV was further studied and compared with other AHSVs.

The VP5 gene was amplified from the AHSV2020/043 sample using conventional gel-based RT-PCR with a set of primers specific to the VP5 gene of the AHSV-1 vaccine strain (GenBank accession no. KT030334, [15]) and the AHSV-1 Thai field strain (GenBank accession no. MT586217, [6]) using PrimerSelect (Lasergene, Madison, WI, USA). The primer sequences are presented in Table 1. The PCR reaction comprised 1 µL of cDNA template, 1× Phusion HF buffer (Thermo Fisher Scientific), 2 mM dNTP mix (Biotechrabbit™), 2 µM forward and reverse primers, 0.2 U/µL Phusion DNA polymerase (Thermo Fisher Scientific), and RNase/DNase-free water to make the volume up to 10 μL. The PCR cycle was performed in a thermal cycler (Mastercycler^®^ nexus GSX1, Eppendorf) with the following thermal profile: 98 °C for 2 min for initial denaturation, then 35 cycles of 98 °C for 10 s, 65 °C for 15 s, and 72 °C for 1 min, followed by a final extension step at 72 °C for 10 min. The 1564 bp amplicons were then ligated to pGEM^®^-T Easy vector (Promega, Madison, WI, USA) and cloned into DH5α competent cells. The plasmid containing VP5 was then submitted for Capillary Electrophoresis Sequencing (CES) (Macrogen, Seoul, Republic of Korea). The DNA sequence was searched for the highest similarity using BLASTn (https://blast.ncbi.nlm.nih.gov/, accessed on 20 April 2020) and subsequently compared with the VP5 gene of AHSV-1 from the Thai field strain, the AHSV-1, 3, and 4 vaccine strains, and reference sequences retrieved from GenBank using MegAlign (Lasergene). Phylogenetic analysis was performed with MEGA-12 software V.11.0.13 (https://www.megasoftware.net/, accessed on 20 April 2020) using the Neighbor-Joining method with 1000 bootstrap replicates and the Tamura–Nei model with a Gamma distribution to find relationships between the VP5 gene of AHSV-1 from the Thai field strain and other AHSV strains.

### 2.5. Type-Specific (VP5) RT Nested PCR and RT Real-Time PCR for AHSV-1 Thai Field Strain and Vaccine Strain Differentiation

To confirmed that the vaccinated horses were AHSV-positive due to only AHSV vaccine strains, type-specific RT nested PCR was used for AHSV strain differentiation, according to a previous report [13]. Briefly, in the first round of nested PCR, positive samples were detected by the first two sets of outer primers, OP1 and OP2 (Table 1). OP1 amplified both the AHSV-1 Thai field strain and the AHSV-1 vaccine strain, giving a 684 bp PCR product. OP2 was specific to AHSV-3 and 4 of the vaccine viruses, giving a 686 bp PCR product. The PCR reactions were set at 25 µL using the same conditions and thermal profile as described above for group-specific (VP7) RT-PCR, with different annealing temperatures (56 °C for OP1 and 45 °C for OP2) for 30 s and an extension temperature of 72 °C for 45 s.

Five microliters of either OP1 or OP2 amplicons were then subjected to the second round of RT nested PCR using the above protocol with the inner primers, comprising four sets of the VP5-specific primers (Table 1). The OP1 amplicon was further amplified with two inner primer sets, ITH1 and IV1, and the OP2 amplicon was used as the template for the other two inner primer sets, IV3 and IV4. PCRs were then performed in a thermal cycler (Mastercycler^®^ nexus GSX1, Eppendorf) with a thermal profile similar to that for the outer primer reaction, but with an annealing temperature of 50 °C for all four inner primer sets. The amplified nested PCR products were confirmed by gel electrophoresis in 1.2% agarose gel. The positive samples from each primer set showed different sizes of PCR products: 547 bp for Thai AHSV-1, and 228, 469, and 437 bp for the AHSV-1, 3, and 4 vaccine components, respectively.

Samples that tested negative with the nested PCR method were tested with VP5-specific RT real-time PCR for AHSV confirmation using the same four inner primer sets used in the nested PCR (Table 1). The RT real-time PCR was performed following the procedure described for the previous VP7-specific RT real-time PCR using iTaqTM Universal SYBR^®^ Green supermix (BioRad, Hercules, CA, USA). The PCR profile was 95 °C for 30 s, followed by 40 cycles of 95 °C for 5 s and 60 °C for 40 s. Finally, melting curve analysis was performed by slowly heating from 65 to 95 °C with 0.5 °C increments for 10 s/step. The positive/negative thresholds were similar to those described for the VP7 RT real-time PCR. The Tm values, measured through melting curve analysis, of each specific primer set were used to confirm the specific amplification products. The Tm value of a single melt peak from the PCR products of the AHSV-1 Thai field strain was 81.50 °C (±0.5), that of the vaccine strain serotype 1 products was 79.50 °C (±0.5), those of the serotype 3 products were 79 °C (±0.5) and 84 °C (±0.5) in double peaks, and that of the serotype 4 products was 83.50 °C (±0.5) in a wide peak.

The results of the VP5-specific RT nested PCR and RT real-time PCR were used in the subsequent viremic-phase study of AHSV-LAV-vaccinated horses to assure the non-infected status of all the vaccinated horses. In accordance with the Thai DLD policy, none of the infected horses were vaccinated; therefore, we did not perform this assay for the 5 naturally infected naïve horses.

### 2.6. Immune Response Against AHSV by Blocking ELISA (bELISA)

The ELISA technique used in this study was competitive blocking ELISA (bELISA). We used a commercial test kit (INgezim AHSV Compac Plus, INGENASA, Madrid, Spain) that can detect antibodies for all AHSV strains in a serum sample using peroxidase-conjugated monoclonal antibodies (Mab) specific to VP7 of AHSV. The bELISA was performed in accordance with the manufacturer’s instructions. The OD value was measured with a microplate reader (BioTek, Winooski, VT, USA) and then used to calculate the blocking percentage (BP). If the BP value of a sample was less than 45%, the sample was considered to be negative or containing no antibodies against VP7 of AHSV. A sample was considered to be positive if its BP value was higher than 50%, meaning that the sample contained antibodies against VP7 of AHSV. Samples with a BP value between 45 and 50% were considered doubtful and required retesting.

### 2.7. Statistics

Descriptive statistics were used to summarize the data. Variations in the percentage of AHSV-positive horses, either by natural infection or vaccination, in each week were calculated using the 95% CI and reported as the standard error of proportions. Antibody and viremia levels are presented as the mean ± standard error (SE).

## 3. Results

### 3.1. Characterization and Phylogenetic Analysis of VP5 Gene

Since sequence alignment of the 10 genomic segments revealed VP5 to be the most genetically distinct segment among the AHSV-1 Thai field and vaccine strains, we investigated this gene in more detail to understand the extent of genetic divergence and its implications for diagnostic applications. Accordingly, an AHSV-1-positive sample, as determined by group-specific (VP7) RT-PCR (Figure S3A), namely AHSV2020/043, which was collected from an infected horse in Chachoengsao province, was used for the VP5 gene study and assay development. The nucleotide sequence of the AHSV2020/043 VP5 gene was confirmed to be 1564 bp in size (Figure S3B) and it was verified by DNA sequencing.

The VP5 nucleotide sequence of AHSV2020/043 has 99.9% similarity with the available sequences of AHSV-1 that emerged in Thailand in 2020 (GenBank accession no. MT586217, MW387431, and MT711963). When compared with the AHSV-1 vaccine strain (GenBank accession no. KT030334), the nucleotide sequence similarity of AHSV1-VP5 of the Thai and vaccine strains is only 85.5% (Table 2). Moreover, the VP5 gene of the other two AHSV serotypes in AHSV-LAV combination 1, AHSV-3 (GenBank accession no. KT030344) and AHSV-4 (GenBank accession no. KT030354), has about 72.4% and 71.1% similarity, respectively, compared to VP5 of Thai AHSV-1, including the AHSV2020/043 isolate (Table 2). The sequence comparison also revealed that the VP5 gene sequence of the Thai field strain is 99–99.5% identical to that of AHSV-2 field strains (GenBank accession no. OM289934, KP009636, FJ196589) present in South Africa.

**Figure 2:**
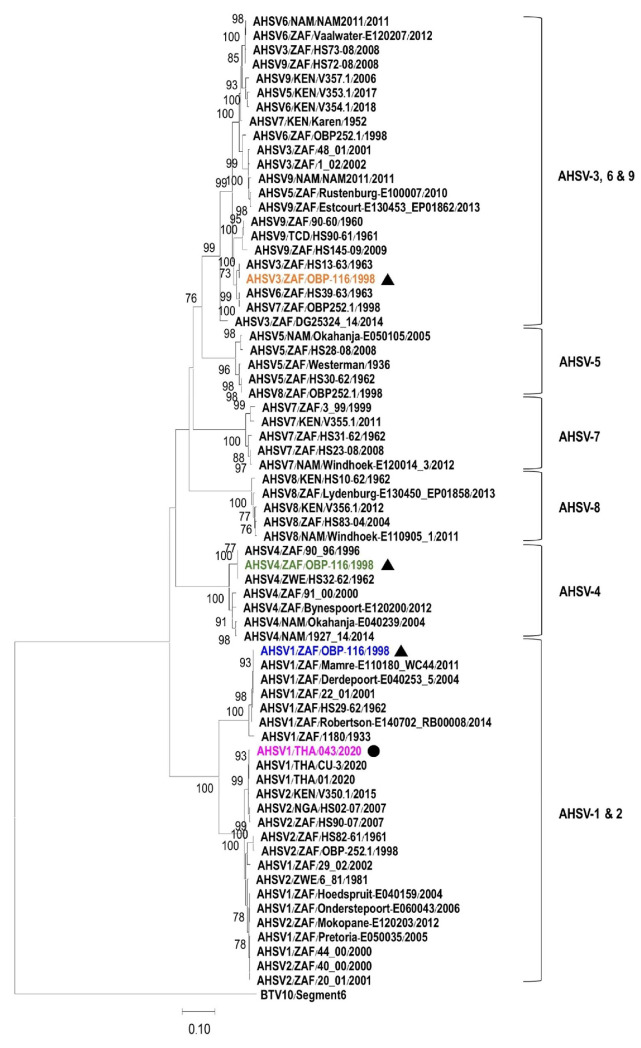
The phylogenetic tree based on the VP5 genes of AHSV serotypes 1–9. The tree was constructed using the Neighbor-Joining method with 1000 bootstrap replicates and the Tamura–Nei method with Gamma distribution, showing the genetic relationship of gene segment 6 (VP5 gene) of AHSV serotypes 1 to 9. The isolate AHSV2020/043 (AHSV1/THA/043/2020) in this study (pink letters) is labeled with a filled circle (●). The viral compositions of the vaccine, including AHSV-1 (purple letters) (GenBank accession no. KT030334), AHSV-3 (orange letters) (GenBank accession no. KT030344), and AHSV-4 (green letters) (GenBank accession no. KT030354), are indicated by filled triangles (▲). The nucleotide sequence of the VP5 gene of bluetongue virus-10 (GenBank accession no. NT006010) served as an outgroup.

Furthermore, a phylogenetic tree was constructed using a complete VP5 nucleotide sequence of AHSV2020/043 and the other 87 nucleotide sequences of AHSV serotypes 1 to 9 available in the GenBank database. The VP5 gene of bluetongue virus-10 (BTV-10) was also included as an outgroup. The tree shows that the VP5 sequences of AHSV-1 and 2 are placed in the same cluster, while the VP5 genes of AHSV serotypes 3, 6, and 9 are grouped together. The VP5 genes of AHSV serotypes 4, 5, 7, and 8 are clustered separately, forming their own groups (Figure 2). The VP5 gene of AHSV2020/043 is closely related to other VP5 genes of Thai AHSV-1, such as AHSV1/THA/CU-3/2020 (GenBank accession no. MW387431) and AHSV1/THA/01/2020 (GenBank accession no. MT586217), but it is distantly related to the VP5 gene of the AHSV-1 vaccine strain (AHSV1/ZAF/OBP-116/1998, GenBank accession no. KT030334), which is separated into a different branch.

### 3.2. Viremic Phase and Clinical Signs of Naturally Infected Naïve Horses

Two farms in Nakhon Ratchasima, designated as A and B, were included in this study (Figure 1 and Figure S1). All of the horses from these farms were confirmed to have AHS status. Farm A had 16 horses, 12 of whom were sick and 8 of whom died, resulting in 66.7% case fatality. Farm B was quite isolated and had only one horse. In addition, Farm C had 146 horses, of whom 100 developed AHS signs and 65 died, resulting in 65% case fatality. All clinically ill horses that survived the outbreak were confirmed to be AHSV-positive by VP7-specific RT-PCR and left unvaccinated, in accordance with the DLD policy, to avoid possible genetic reassortment between vaccine and field strains. It is important to note that we collected blood samples according to our plan; however, Farm C was not included in the viremia study because the collected EDTA samples perished, leading to only 5 of the 15 unvaccinated horses being included in the AHSV kinetics study. However, the kinetics of the immune response of infected horses on Farm C could be investigated as originally planned. These horses were kept in insect-proof stables to prevent further exposure to infected vectors and re-infection. Based on clinical sign observation, all four horses from Farm A showed clinical signs of AHS, including fever, depression, anorexia, edema of the supraorbital fossa, conjunctival congestion, and dyspnea, and had viremia for the first 2–3 weeks of infection. AHSV was detected in the horses’ blood 4–5 days before they showed clinical signs of AHS, and viremia and illness disappeared at the same time in week 3 after the disease’s onset. One of the horses on this farm was AHSV-positive from week 1 to week 5. Farm C had been confronted with an AHS outbreak for 3–4 weeks before we approached the farm. Eight horses on Farm C were found to be sick and AHSV-positive when we first observed the horses at 3–4 weeks post-outbreak. According to farm records, specific clinical manifestations were not documented. Two horses on this farm and one on Farm B showed no signs of illness throughout the study period. The virus presented in the horse from Farm B for a week, while the other two horses in Farm C exhibited viremia until weeks 3 and 4 after the outbreak.

**Figure 3:**
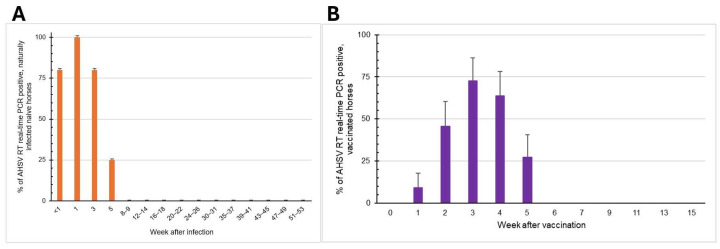
The percentage of AHSV-positive horses determined weekly after infection. AHSV was detected using group-specific (VP7) RT real-time PCR in naturally infected horses after infection (**A**) and in AHSV-LAV combination-1-vaccinated horses before and after vaccination (**B**). On the Y-axis, <1 indicates day 2 or 3 post-infection. On the X-axis, 0 (**B**) represents day 0, which is the pre-vaccination date. The bars represent the standard error of proportions.

The data of all the infected horses in Farms A and B were compiled, and the percentage of infected horses was plotted over time (Figure 3A). As determined by both the VP7-specific RT-PCR and RT-real-time PCR assays, viremia was found within a week post-infection in 4/5 horses (80%), at week 1 in 5/5 horses (100%), at week 3 in 4/5 horses (80%), and at week 5 in 1/4 horses (25%). From weeks 8–9 post-infection onward, AHSV was not detected in infected horses. In conclusion, the virus presented in the blood of naturally infected horses from 1 to 5 weeks, with the average viremic phase of 3 weeks.

### 3.3. Viremic Phase of AHSV-LAV Combination-1-Vaccinated Horses

**Figure 4:**
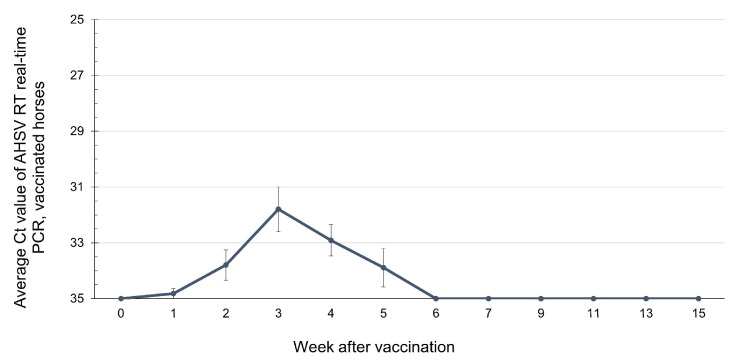
The average Ct values of AHSV-LAV in vaccinated horses, detected weekly by group-specific (VP7) RT real-time PCR before and after vaccination. The positive and negative cutoffs are at the Ct values < 35 and ≥35, respectively. On the X-axis, 0 represents day 0, which indicates the pre-vaccination serum sample. The bars in the plot represent the standard error.

AHSV VP7-positive samples from horses immunized with AHSV-LAV were further tested for the AHSV-1 Thai field strain and vaccine strain differentiation using type-specific (VP5) RT nested PCR and real-time PCR. The results revealed that the blood samples from 11 horses that tested positive for AHSV VP7 were confirmed to contain only vaccine strain, and their serotypes could be identified. AHSV was detected at week 1 post-vaccination in 1/11 horses (9.09%), at week 2 in 5/11 horses (45.45%), at week 3 in 8/11 horses (72.72%), at week 4 in 7/11 horses (63.63%), and at week 5 in 3/11 horses (27.27%) (Figure 3B). During weeks 6–15 post-vaccination, all horses were negative for AHSV. Among all 11 horses, only one showed negative AHSV results throughout the study period. When the average Ct values from blood collected between weeks 1 and 5 in this group were plotted over time, a low level of viremia appeared at the first week post-vaccination, and the level peaked at week 3 (Figure 4). However, none of these horses showed any signs of illness or allergic reactions throughout the study period after vaccination.

### 3.4. Detection of Immune Responses Against AHSV Using bELISA

**Figure 5:**
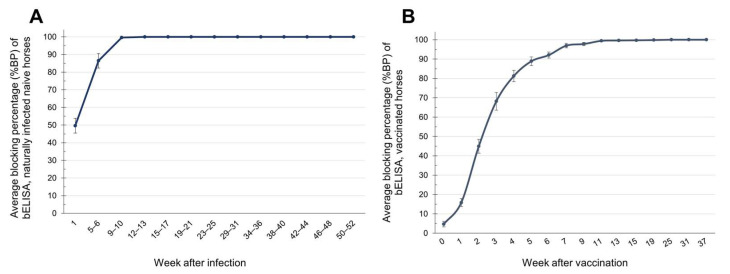
The kinetics of antibody responses against AHSV. %BP indicates the blocking percentage obtained from bELISA of serum samples from naturally infected naïve horses (**A**) and AHSV-LAV combination-1-vaccinated horses (**B**). The graphs plot the average %BP with standard error against the time in weeks after infection (**A**) and vaccination (**B**). A %BP value > 50 is positive, a value < 45 is negative, and a value ≥ 45 and ≤50 is doubtful. The bars in the plots represent the standard error.

The results for the immune response against AHSV, obtained using bELISA, in naturally infected, naïve horses showed that 6 out of 15 horses (46.15%) had positive results in the first week after infection, but the average %BP value remained doubtful at 49.61 ± 4.2 (*SE*). The average %BP increased to 86.45 ± 4.1 (*SE*), with 11 horses (73.33%) showing seroconversion, in weeks 5–6, and the average %BP rose to 99.65 ± 0.3 (*SE*) by weeks 9–10. Subsequently, in all horses, the antibody level reached a BP value of 100% and remained at this level until week 50–52 (100%), which was the end of the study period (Figure 5A).

In AHSV-LAV combination-1-vaccinated horses, it was found that by the second week after vaccination, 4 out of 11 horses (36.36%) had seroconverted against AHSV and exhibited low antibody levels. As a result, the average %BP value of all 11 horses remained negative at 44.9%. By week 3, 10/11 horses (90%) were immune, with an average %BP value of 68.14 ± 4.6 (*SE*). By week 4, all horses (100%) had an average %BP value that had increased to 81.23 ± 2.9 (*SE*). The immunity level continued to rise, reaching an average BP value of 100% by week 25 and remaining at this level until week 37, when the study was terminated due to the boosting vaccination program (Figure 5B).

## 4. Discussion

African horse sickness (AHS) was first confirmed and caused a rapidly spreading outbreak in Thailand in March 2020. Whole-genome sequencing revealed that the causative virus was a serotype 1 phylogenetically related to a South African isolate [6]. In our study, the VP5 nucleotide sequence amplified from a blood sample collected from a sick horse (AHSV2020/043) in Chachoengsao province was similar to the VP5 sequences of other Thai AHSV isolates, including TH2020/01 (GenBank accession no. MT586214), 1/E.caballus/THA/2020/CU-3 (GenBank accession no. MW387431), and 110983/63-Thailand-NParks-SG (GenBank accession no. MT711963) [6,7,16], present during the first AHS outbreak in Nakhon Ratchasima province in Thailand. This result confirms that the sick horse was infected with the AHSV strain that emerged in Thailand in 2020. Furthermore, we found that the VP5 nucleotide sequence of 2020 Thai AHSV-1 was quite different from that of the AHSV-1 vaccine strain (GenBank accession no. KT030334). Based on the nucleotide sequence alignment of all ten genomic segments (segments 1–10) of the Thai AHSV-1 field strain compared to the corresponding genes of the serotype 1 vaccine strain, segment 6 (VP5 gene) exhibited the greatest genetic divergence. According to a previous study, the 3′ end of the VP5 gene showed notably low similarity between the Thai field strain and the serotype 1 vaccine strain (1/Labstr/ZAF/1998/OBP-116) (GenBank accession no. KT030334) [17]. Surprisingly, based on comparison of the VP5 nucleotide sequences of AHSVs in the GenBank database using Nucleotide Blast (blast.ncbi.nlm.nih.gov), the VP5 gene of our Thai field strain was 99.5% similar to the AHSV serotype 2 isolate V350.1 found in Kenya in 2015. In addition, VP5 genes of Thai AHSV-1 isolates including TH2020/01, 110983/63-Thailand-NParks-SG and AHSV2020/043 in this study were at least 99% identical to that of AHSV-2 found in South Africa in 2007 and in Nigeria in 2008 [6,16]. This observation may reflect possible evolutionary events, such as genetic reassortment or recombination between serotypes. Further surveillance and sequence analysis of circulating strains will be needed to clarify the significance of this observation.

Our study of the dynamics of viral infection and the immune response against AHSV in naturally infected and vaccinated animals revealed that most of the naturally infected horses exhibited viremia within the first week, which peaked at week 1 and persisted for up to 5 weeks after infection. Normally, the viremic period in AHSV-infected horses is 4–8 days, but it can extend for up to 21 days [9]. The long viremic period of 3–5 weeks observed in this study might be the consequence of various factors, including the environment, weather conditions, and horse care protocol at each horse farm. These factors can affect the amount of insect vectors, which directly correlates with the number of viruses present, virus transmission, and the severity of the AHS disease [18]. Despite the installation of insect-proof net stables with and without insecticide treating, a low risk of horses being bitten by the vectors inside the housing remained [19]. In our study, environmental risk factors might have played a partial role in the longer viremia observed in the horses on Farm A, as Farm A was surrounded with dense layers of trees and had a canal running alongside it. One horse on Farm A that exhibited viremia for up to 5 weeks showed slow development of an immune response against the virus, which might have caused slow virus elimination and prolonged viremia. On the other hand, Farm B was quite isolated, and the farmer started insect vector control and prevention early after noticing the nearby AHS outbreak. The horse on this farm was exposed to few vectors with a low viral load, resulting in it showing no clinical signs after infection and rapid virus elimination after immune development. The viremic phase in the studied horse from this farm lasted for only a week. However, only five naturally infected horses in total were included in the viremia analysis. This limited sample size restricts the ability to generalize the finding of AHSV being detectable for up to 5 weeks post-infection. Nevertheless, our data provide rare longitudinal evidence of viremia persistence during the first AHS outbreak in Thailand and Southeast Asia.

Our study of the dynamics of the immune response against AHSV in naturally infected naïve horses and AHSV-LAV combination-1-vaccinated horses revealed that seroconversion began after the first and second weeks, respectively. Additionally, the immune response that developed in vaccinated horses was more homogeneous than that in naturally infected horses. In previous studies, however, AHSV-LAV-immunized horses developed antibody titers, as tested by indirect ELISA, in week 2 after immunization. The viremic phase was found during days 7–14, prior to the development of the immune response [10]. Another study on horses immunized with AHSV-LAV found that their antibody titers against the VP7 gene of AHSV, as tested by bELISA, were positive from days 21–30 after the first vaccination [20]. In addition, our study revealed that the immune response against AHSV lasted for at least a year in naturally infected horses and for at least 37 weeks (8–9 months, which was the end of the study) in vaccinated horses kept in stables. These results show agreement with a previous study in which the antibody titers of horses vaccinated with AHSV-LAV combination 1 were found to positive, according to bELISA, from 1 to 12 months [12]. Although bELISA provides a simpler and more practical approach to detecting antibody levels in horses, the immunity described here refers to total antibodies, which may not directly correlate with protective immunity as measured by the serum neutralization test (SNT) [10]. Therefore, future studies should incorporate the SNT to better assess functional protective immunity against AHSV in both naturally infected and vaccinated horses. In a previous study, three out of six vaccinated horses developed neutralizing antibody titers against AHSV-1 from week 4 after vaccination with combination-1 of AHSV-LAV, while the remaining horses were found to be positive at week 8 after vaccination [11]. Another study reported that all 11 vaccinated horses from two groups became SNT-positive at week 8 after vaccination with AHSV-LAV combination-1 at week 0 and 16 and combination-2 at week 4 and 24. Their immunity remained positive until week 16, when the horses were re-vaccinated with the same vaccine [21]. Although our study demonstrated antibody persistence for at least 37 weeks in vaccinated horses, the true duration of immunity remains uncertain, since the study was terminated due to the implementation of a national booster program. Even if antibodies persist for up to 12 months or longer, this does not necessarily ensure protective activity in every horse [10]. Therefore, annual vaccination of horses is required to maintain protective levels against field viruses in order to reduce losses from AHS [22], as non-vaccinated horses have an 8.7-times-higher risk of death from AHS than vaccinated horses [23].

## 5. Conclusions

The control and prevention of AHS outbreaks are crucial and require the implementation of practices to reduce the risks and prevent the spread of AHSV, particularly in countries that have never experienced this disease and have highly susceptible horse populations. One important factor to consider is insect vectors, which can carry AHSV from an infected horse to uninfected horse. Stabling horses that live in an outbreak or risk area, whether they are infected or vaccinated, with an insect-proof net treated with insecticide or insect repellent can help to reduce the possibility of horse exposure to vectors. The length of time for which horses should be housed depends on their viremic duration and antibody levels against AHSV, which should increase to a protective level in both infected and vaccinated horses. We found that a high level of antibody titers against AHSV developed by 3–5 weeks after infection and vaccination, and remained positive for at least 1 year in naturally infected naïve horses and 37 weeks in AHSV-LAV combination-1-vaccinated horses. The virus was detectable up to week 5 after infection and vaccination. Thus, horses should be stabled with an insect-proof net for 5 weeks or 35 days to decrease the risk of AHSV transmission. Vector control practices should also be implemented concurrently. In addition, vaccination of horses is an essential measure to control and prevent AHS transmission.

## Figures and Tables

**Table 1:** The primers used in this study.

Primers	Sequences (5′ → 3′)	Purpose	Target	PCR Product Size (bp)
VP7_F	CGC-GAT-AGC-AGC-AAG-AGC-C	Group-specific amplification	VP7	256
VP7_R	GTT-GCC-AAC-GCC-TGA-TCA-TA			
VP5_F	GTT-TAT-TTT-TCC-AGA-AGC-CAT-GGG-TAA-ATT-C	Cloning	VP5	1564
VP5_R	GTA-AGT-GTT-TTT-CCC-GCC-CAC-AGG-CTC-C			
OP1_F	TCG-CAT-CTC-AAG-GTT-GC	First round of nested PCR (outer primers)	VP5 genes of AHSV-1	684
OP1_R	AAG-CGC-GTT-CAT-TAT-CGT-CC			
OP2_F	TAC-GTM-GAA-AAA-GCG-CT	First round of nested PCR (outer primers)	VP5 genes of AHSV-3 and -4	686
OP2_R	TGA-TGA-TGC-GGY-GCA-ATG			
ITH1_F	GCT-AGC-GGT-TGC-AAT-CAA-GTC-AAA-G	Second round of nested PCR (inner primers)	VP5 genes of Thai AHSV-1	547
ITH1_R	CAG-ATC-TGT-GTT-ATG-CAC-CAG-CTG-TAG-T			
IV1_F	ATA-TAA-TGC-ATG-GGG-GTG-CTG-TT	Second round of nested PCR (inner primers)	VP5 genes of AHSV-1 vaccine strain	228
IV1_R	GGA-GAT-CAG-TAT-TAT-GAA-CCA-ACT-GTA-AA			
IV3_F	CCT-CCA-AAC-GGA-AGA-GGA-TTT-AAG-AAC-TTC	Second round of nested PCR (inner primers)	VP5 genes of AHSV-3 vaccine strain	469
IV3_R	TTC-GTA-TTC-CTT-CTT-CAC-TAG-AGG-CAT-G			
IV4_F	GTT-ACA-AAC-AGA-GGA-AGA-TTT-GCG-GAC-ACG	Second round of nested PCR (inner primers)	VP5 genes of AHSV-4 vaccine strain	437
IV4_R	CAT-CGA-TTA-CGT-GCT-GCG-TTT-CTA-CG			

**Table 2:** The similarity between VP5 of AHSV2020/043 and other VP5 sequences from Thai AHSV-1, the AHSV-2 field strains, and AHSV components of AHSV-LAV combination 1.

Strain	Accession no.	Location	Year	%Similarity to AHSV2020/043 VP5
Field AHSV-1	MT586217	Thailand	2020	99.9
Vaccine AHSV-1	KT030334	South Africa	1998	85.5
Vaccine AHSV-3	KT030344	South Africa	1998	72.4
Vaccine AHSV-4	KT030354	South Africa	1998	71.1
Field AHSV-2	OM289934	Kenya	2015	99.5
Field AHSV-2	KP009636	South Africa	2007	99.1
Field AHSV-2	FJ196589	Nigeria	2008	99

## Data Availability

Most data are included in this manucscript. However, the data of RT Nested-PCR were presented at the 63th KU conference and it considered as a published data.

## Supplementary Materials

The following supporting information can be downloaded at https://www.mdpi.com/article/10.3390/vetsci12090878/s1, Figure S1: A map of Nakhon Ratchasima province and the locations of Farms A, B, and C in Pakchong district. Farm A is 10 km and 13 km from Farms B and C, respectively. The distance between Farms B and C is 18 km; Figure S2: The timeline of the Thailand AHS outbreak with confirmed cases, sample collection, and vaccination of horses. Vaccination was conducted only in AHS-free horses. Samples were collected from Farms A, B, C, and D. Horses that were confirmed to be AHSV positive by RT-PCR and serological analyses were not vaccinated throughout the study, in accordance with the DLD policy to avoid genetic reassortment between field and vaccine strains; Figure S3: Agarose gel electrophoresis photograph showing AHSV-VP7 (A)- and AHSV-VP5 (B)-positive PCR products. Lane 1: negative control; lane 2: marker; lane 3: AHSV2020/043 sample; lane 4: positive control (AHSV-LAV).

## Abbreviations

The following abbreviations are used in this manuscript:

AHS	African Horse Sickness
AHSV	African Horse Sickness Virus
bELISA	Blocking ELISA
BLASTn	Nucleotide BLAST
bp	Base Pair
BP	Blocking Percentage
cDNA	Complementary Deoxyribonucleic Acid
CES	Capillary Electrophoresis Sequencing
Ct	Cycle Threshold
DLD	Department of Livestock Development
DNA	Deoxyribonucleic Acid
DNase	Deoxyribonuclease
dNTP	Deoxyribonucleotide Triphosphate
EDTA	Ethylenediaminetetraacetic Acid
ELISA	Enzyme-linked Immunosorbent Assay
KVDC	Kamphaeng Saen Veterinary Diagnostic Center
LAV	Live attenuated Vaccine
Mab	Monoclonal Antibody
NIAH	National Institute of Animal Health
NS	Viral Non-structural Protein
OBP	Onderstepoort Biological Products
OD	Optical Density
PCR	Polymerase Chain Reaction
RFU	Relative Fluorescence Unit
RNA	Ribonucleic Acid
RNase	Ribonuclease
Rpm	Revolutions Per Minute
RT	Reverse Transcription
RT-PCR	Reverse Transcription Polymerase Chain Reaction
SNT	Serum Neutralization Test
Tm	Melting Temperature
U	Unit
VP	Viral Structural Protein
WOAH	World Organisation for Animal Health
°C	Degree Celsius
mM	Millimolar
ml	Milliliter
µL	Microliter
μM	Micromolar
