# Supplementary material for: The Viremic Phase and Humoral Immune Response Against African Horse Sickness Virus That Emerged in Thailand in 2020
Source: Vet Sci. 2025 Sep 11;12(9):878. doi: 10.3390/vetsci12090878 (PMC12474096; doi:10.3390/vetsci12090878)
Supplement: Supplementary file 1 [file vetsci-12-00878-s001.zip › Supplementary Figures.pdf]

Supplementary Figures

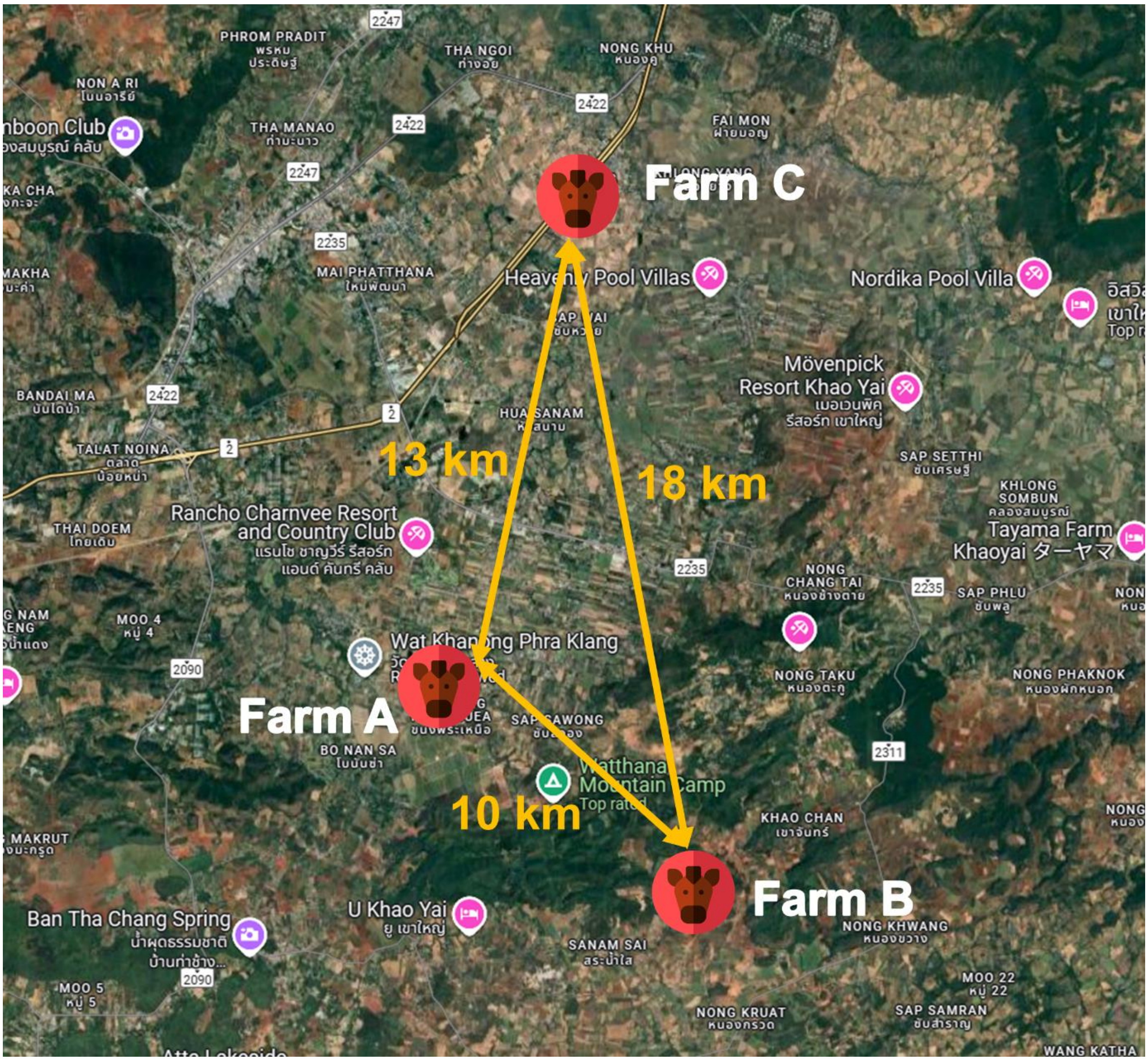

Figure S1 A map of Nakhon Ratchasima province and the locations of Farms A, B, and C in Pakchong district. Farm A is 10 km and 13 km from Farms B and C, respectively. The distance between Farms B and C is 18 km.

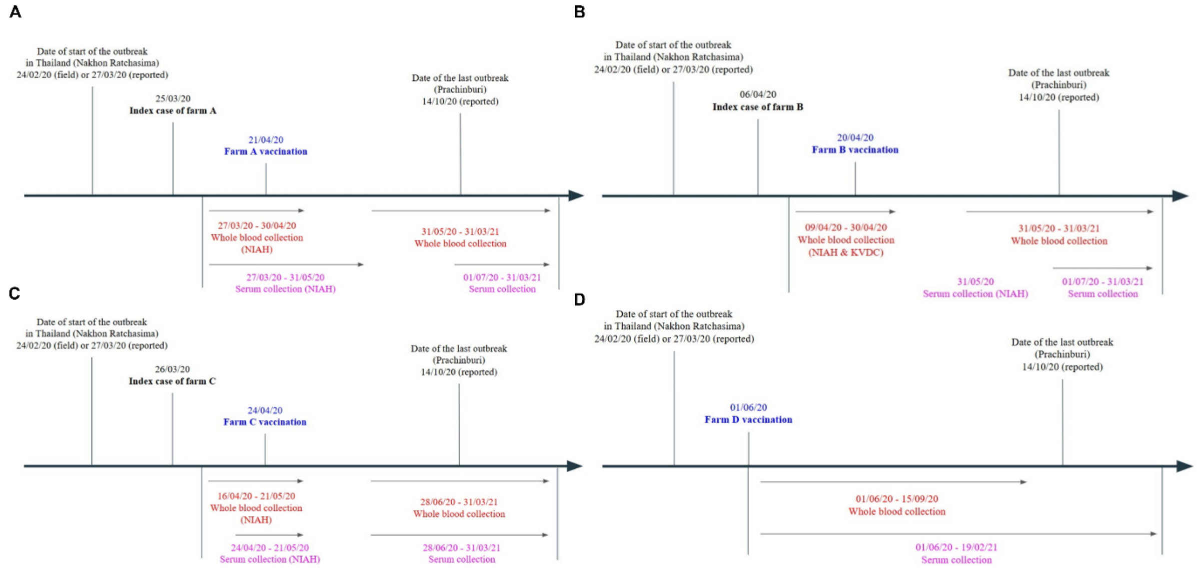

Figure S2: The timeline of the Thailand AHS outbreak with confirmed cases, sample collection, and vaccination of horses. Vaccination was conducted only in AHS-free horses. Samples were collected from Farms A, B, C, and D. Horses that were confirmed to be AHSV positive by RT-PCR and serological analyses were not vaccinated throughout the study, in accordance with the DLD policy to avoid genetic reassortment between field and vaccine strains.

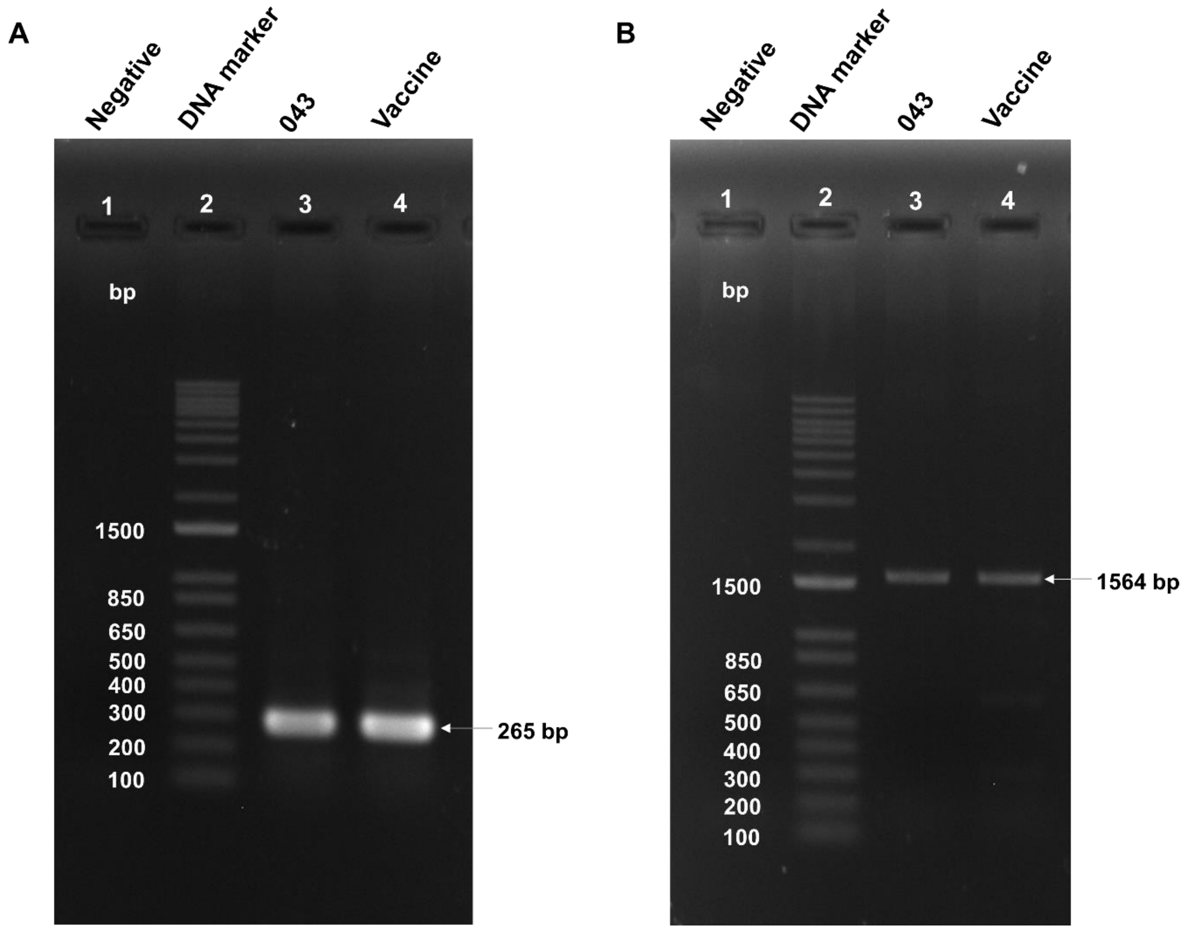

Figure S3: Agarose gel electrophoresis photograph showing AHSV-VP7 (A)- and AHSV-VP5 (B)-positive PCR products. Lane 1: negative control; lane 2: marker; lane 3: AHSV2020/043 sample; lane 4: positive control (AHSV-LAV).
